# ACE: an efficient and sensitive tool to detect insecticide resistance-associated mutations in insect acetylcholinesterase from RNA-Seq data

**DOI:** 10.1186/s12859-017-1741-6

**Published:** 2017-07-10

**Authors:** Dianhao Guo, Jiapeng Luo, Yuenan Zhou, Huamei Xiao, Kang He, Chuanlin Yin, Jianhua Xu, Fei Li

**Affiliations:** 1 Ministry of Agriculture Key Lab of Molecular Biology of Crop Pathogens and Insects, Institute of Insect Science, Zhejiang University, 866 Yuhangtang Road, Hangzhou, 310058 China; 2 Department of Entomology, College of Plant Protection, Nanjing Agricultural University, Nanjing, 210095 China; 3 College of Computer Science and Technology, Nanjing Normal University, Nanjing, 210023 China; 4 College of Life Sciences and Resource Environment, Yichun University, Yichun, 336000 China

**Keywords:** RNA-Seq data, Insecticide resistance, Mutations, Ace, Detection

## Abstract

**Background:**

Insecticide resistance is a substantial problem in controlling agricultural and medical pests. Detecting target site mutations is crucial to manage insecticide resistance. Though PCR-based methods have been widely used in this field, they are time-consuming and inefficient, and typically have a high false positive rate. Acetylcholinesterases (Ace) is the neural target of the widely used organophosphate (OP) and carbamate insecticides. However, there is not any software available to detect insecticide resistance associated mutations in RNA-Seq data at present.

**Results:**

A computational pipeline ACE was developed to detect resistance mutations of *ace* in insect RNA-Seq data. Known *ace* resistance mutations were collected and used as a reference. We constructed a Web server for ACE, and the standalone software in both Linux and Windows versions is available for download. ACE was used to analyse 971 RNA-Seq data from 136 studies in 7 insect pests. The mutation frequency of each RNA-Seq dataset was calculated. The results indicated that the resistance frequency was 30%–44% in an eastern Ugandan *Anopheles* population, thus suggesting this resistance-conferring mutation has reached high frequency in these mosquitoes in Uganda. Analyses of RNA-Seq data from the diamondback moth *Plutella xylostella* indicated that the G227A mutation was positively related with resistance levels to organophosphate or carbamate insecticides. The wasp *Nasonia vitripennis* had a low frequency of resistant reads (<5%), but the agricultural pests *Chilo suppressalis* and *Bemisia tabaci* had a high resistance frequency. All *ace* reads in the 30 *B. tabaci* RNA-Seq data were resistant reads, suggesting that insecticide resistance has spread to very high frequency in *B. tabaci.*

**Conclusions:**

To the best of our knowledge, the ACE pipeline is the first tool to detect resistance mutations from RNA-Seq data, and it facilitates the full utilization of large-scale genetic data obtained by using next-generation sequencing.

**Electronic supplementary material:**

The online version of this article (doi:10.1186/s12859-017-1741-6) contains supplementary material, which is available to authorized users.

## Background

Insect pests are closely connected to human affairs, and they damage approximately one third of the agricultural, forestry and livestock production worldwide and consume tens of billions of dollars annually [1]. Although several alternative strategies such as transgenic crops and biological control measures have recently been implemented in pest control, the use of chemical insecticides remains the most efficient and economic approach. However, use of insecticides has led to resistance, which is one of the best examples of rapid micro-evolution and has challenged the application of insecticides [2, 3]. The study of insecticide resistance is important because of its relevance to food safety, ecological safety and environmental pollution.

Target insensitivity is one of the main mechanisms conferring insecticide resistance. Because of long-term selection by insecticides, mutations are introduced into the active sites of genes that encode proteins that are the targets of insecticides. Given that the mutation frequency in the field population is a reliable indicator of the resistance level, monitoring resistance mutations in a field population of insect pests is highly important [4]. PCR-based methods such as PCR amplification of specific alleles (PASA) [5] and PCR-RFLP [6] are classical approaches that have been widely used. However, PCR-based methods have some disadvantages such as they are time-consuming and inefficient [7–10].

Acetylcholinesterases (*ace*, EC 3.1.1.7) are the target of OP and carbamate insecticides, which have been used to control nearly all notorious agricultural and medical pests such as rice stem borers, Colorado potato beetles, mosquitoes and houseflies. Two *ace* which encoding different ACHE proteins have been found in all insects except the Cyclorrhapha suborder of Diptera [11]. The mutation of *ace* to an insensitive form has been demonstrated as an important mechanism for insecticide resistance in many pests. In *Drosophila melanogaster*, 4 point mutations (F115S, I199V, G303A, and F368Y) have been identified to confer insecticide resistance [12]. Five mutations (V180 L, G262A, G262 V, F327Y, and G365A) in the *ace* of the housefly, *Musca domestica*, either singly or in combination, confer different levels of insecticide resistance [13]. The G119S mutation, which lies within the active “gorge” in *ace-1* of *Anopheles gambiae* and *Culex pipiens*, results in resistance to propoxur [14]. Many resistance-associated mutations have also been identified in other insect pests [15–17].

RNA sequencing (RNA-Seq) provides the whole transcriptome of a biological sample at a given time by using a shotgun strategy with next-generation sequencing (NGS) techniques. The raw reads of the RNA-Seq data contain information on transcript abundance, alternative splicing and single nucleotide polymorphisms (SNP)/mutations [18, 19]. RNA-Seq data are useful in studying insecticide resistance, but unfortunately are not fully utilized at present. Most RNA-Seq data are used as a resource to obtain gene sequences. Here, to fully use RNA-Seq data to study insecticide resistance, we developed a pipeline, ACE, to detect resistance-associated mutations in *ace* genes from RNA-Seq data and applied this pipeline to estimate the mutation frequencies in 7 important insect pests.

## Results

### Evolution analysis of two *ace* genes in insects

**Fig. 1:**
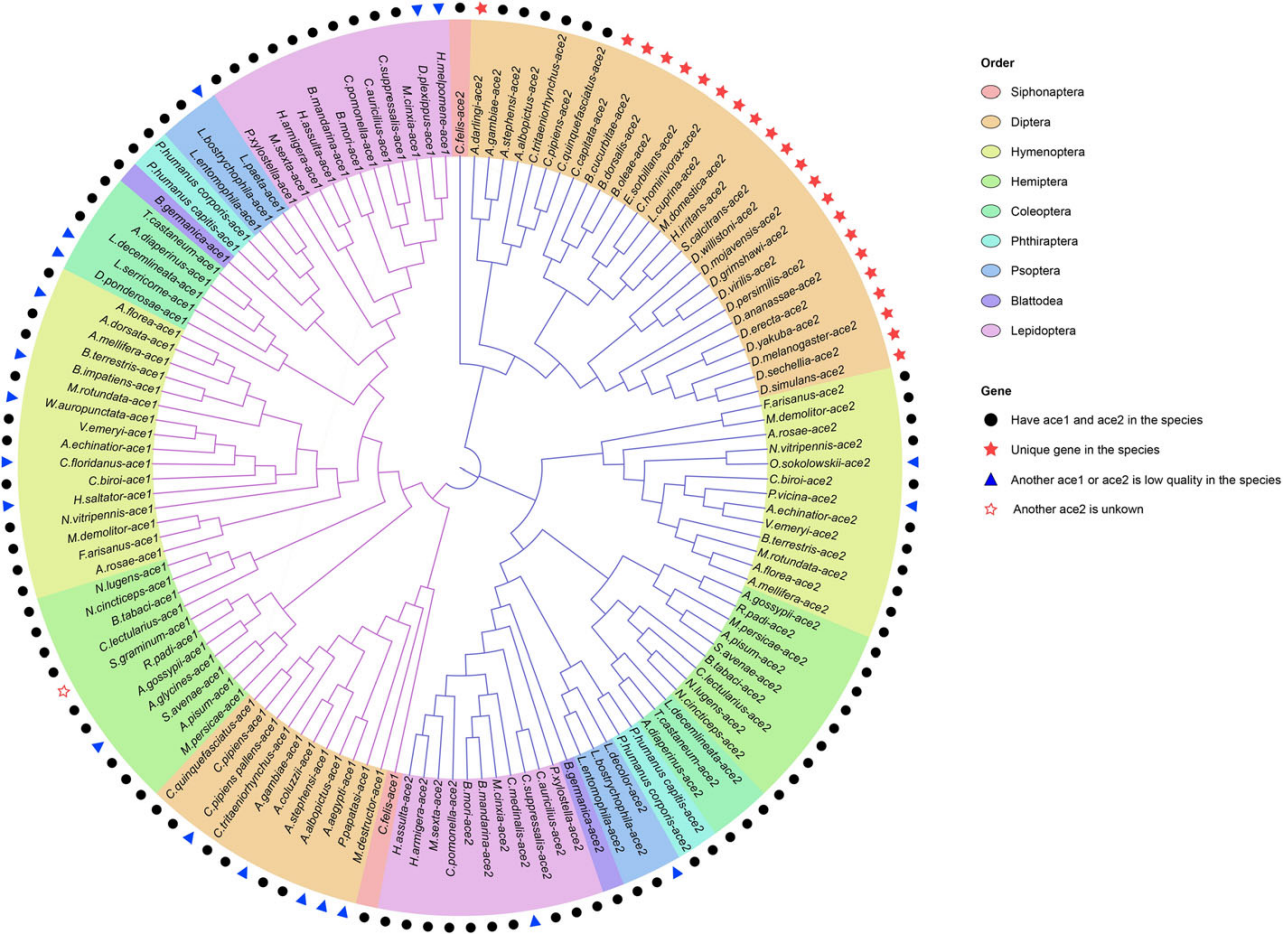
Evolution analysis of two *ace* genes in insects. The amino acid sequences were used for the phylogenetic analysis. The sequence accession numbers are given in Table S1. The neighbour-joining method was used with 1000 replicates. The evolution tree indicated that most insects have two *ace* genes, except for the Cyclorrhapha suborder of Diptera

By searching against the GenBank database and using BLASTP against the InsectBase database with 15 known ACHE protein sequences, we collected 62 *ace1* from 62 species and 70 *ace2* from 70 species. These *ace* genes were from 9 orders, including Siphonaptera, Diptera, Hymenoptera, Hemiptera, Coleoptera, Phthiraptera, Psocoptera, Blattodea and Lepidoptera (Additional file 1: Table S1). To the best of our knowledge, this is the most comprehensive list of insect *ace* to date. Phylogenetic analysis using the neighbour-joining method indicated that most insects have two *aces*, except for the Cyclorrhapha suborder of Diptera (Fig. 1), suggesting that suggesting two *ace* were present before the diversification of insects. The loss of *ace1* occurred in some Diptera insects.

### Insecticide resistance-associated mutations of *ace*

**Fig. 2:**
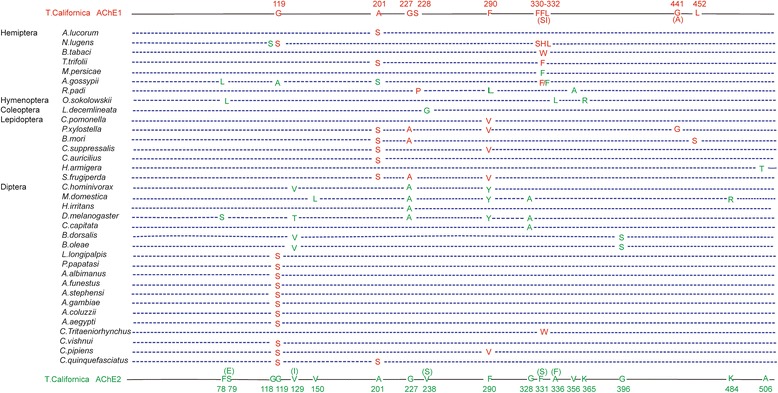
Resistance mutation profile of insect acetylcholinesterases. The mutations were collected from 440 published references. Insect AChEs were aligned with *Torpedo californica* AChE (PDB ID code 1EA5) and the corresponding position of each mutation in *Torpedo* AChE was determined

We performed reference mining from 440 references to obtain a full list of insecticide resistance mutations of the *ace* in insects. Insect ACHEs were aligned with *Torpedo californica* ACHE (PDB ID code 1EA5), and the corresponding position of each mutation in *T. californica ace* was determined. In total, 14 mutations were found at 10 positions in *ace1*, and 22 mutations were found at 18 positions in *ace2* (Fig. 2, Additional file 2: Table S2). Although there were several resistance mutations in both *ace*, most of the mutations occurred at 5 positions, 119, 201, 227, 290 and 331. These positions fall within the active gorge of ACHE, thus demonstrating a common mechanism conferring insecticide resistance.

### ACE pipeline to detect resistance mutations of *ace* genes

**Fig. 3:**
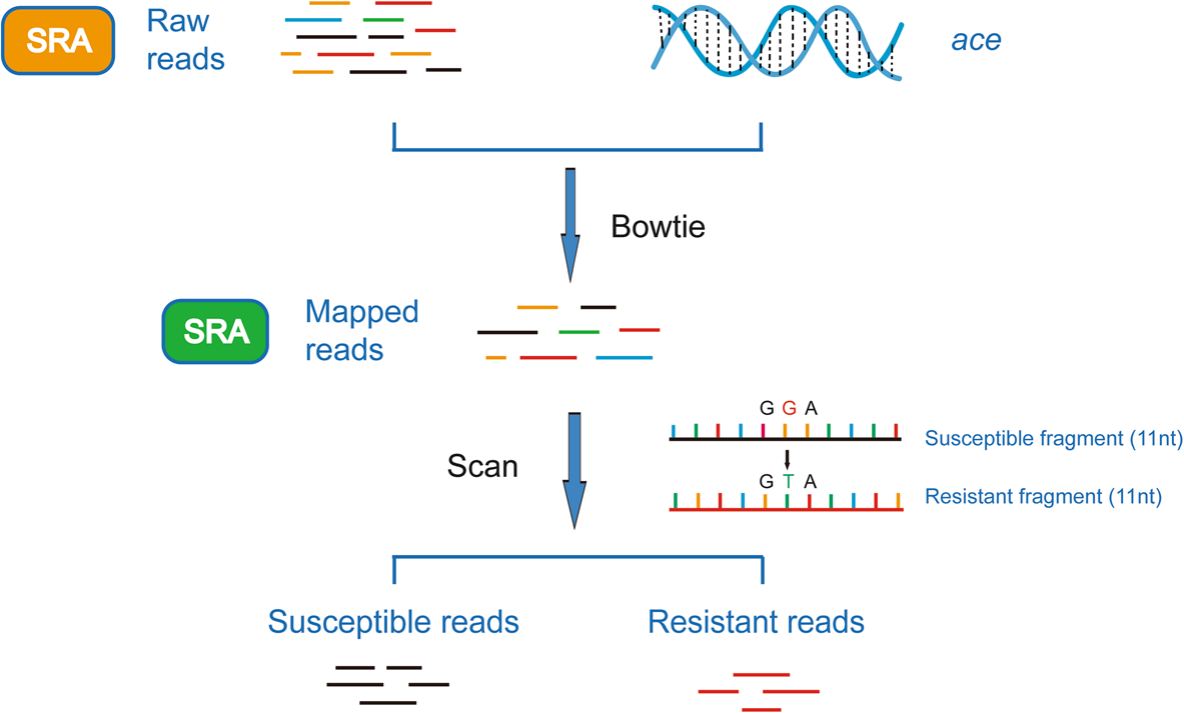
The principles of the ACE pipeline. Raw reads were mapped with insect ace gene sequences by using Bowtie 2. Then, the resistant fragment (11 nt) and susceptible fragment (11 nt) flanking the mutation sites were used to scan the reads mapped with the insect ace gene. The scanned reads were divided into two types: resistant reads and susceptible reads

We developed a pipeline, named ACE, to detect insecticide resistance mutations from RNA-Seq data (Fig. 3). First, the clean reads of the RNA-Seq data (Base-calling quality, Q30 ≥ 85%) in standard Fastq format were mapped against the *ace1* or *ace2* of the species of interest by using Bowtie 2 with the default parameters [20]. This step identified all reads corresponding to *ace*. Second, we constructed a mutation site profile of *ace* for each insect, which consisted of susceptible and resistant fragments (11 nucleotides in length) covering each mutation site. We determined the cutoff of 11 bp based on a pilot survey. If we use a long segment of >13 bp, some reads will be lost. However, if we used a short segment <9 bp, it will be mapped to other non-ace transcripts. Third, the reads that mapped to *ace* were used to scan for susceptible and resistant fragments with a customized Perl script. The reads containing susceptible fragments were treated as susceptible reads, and those containing resistant fragments were resistant reads. The percentages of susceptible or resistant reads were then calculated.

$$ \mathit{\mathsf{Resistance}}\ \mathit{\mathsf{frequency}}=\frac{\mathit{\mathsf{Count}}\ \mathit{\mathsf{of}}\ \mathit{\mathsf{resistant}}\ \mathit{\mathsf{reads}}}{\mathit{\mathsf{count}}\ \mathit{\mathsf{of}}\ \mathit{\mathsf{resistant}}\ \mathit{\mathsf{reads}}+\mathit{\mathsf{count}}\ \mathit{\mathsf{of}}\ \mathit{\mathsf{susceptible}}\ \mathit{\mathsf{reads}}} $$

### Implementation

We developed a standalone software and a Web server for the ACE pipeline. The standalone software is available for download. The Web server can be accessed at http://genome.zju.edu.cn/software/ace/. The Apache HTTP server was deployed in a Red Hat 6.5 Linux operating system. The Web pages were written by using HTML and Cascading Style Sheets (CSS). We also used Asynchronous JavaScript and XML (AJAX) to achieve some of the dynamic parts of the Web pages. The PHP script calls the ACE program, which runs online when the HTTP server receives the request from a Web client. The standalone version was built on the ultrafast short read mapping program Bowtie 2 [21]. All parameters were set as the default except using “--no-unal” as an additional parameter. Both Linux and Windows versions of the ACE standalone software are available. ACE is rapid and took only 5 min to process the 5 Gb RNA-Seq data on a Red Hat server (Dell X3250, Red Hat 6.5 Linux 64 bits, 3.1 GHz 4 CPU each with 4 cores, 32 G memory).

### Application of ACE to analysis of RNA-Seq data in 7 insect pests

**Fig. 4:**
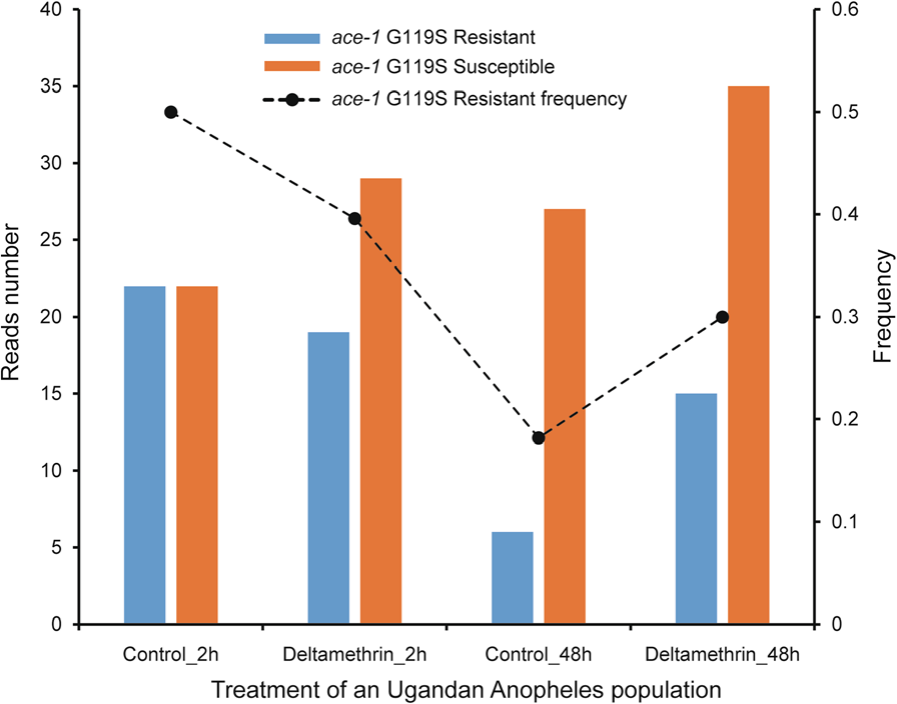
The resistance frequency of four samples of a Ugandan population of *Anopheles gambiae*. The control was an untreated population which has high resistance to pyrethroids. The other two groups were treated with deltamethrin at 2 h or 48 h post treatment. The G119S mutation of *ace1* was detected. The results indicated that the resistance level in this Ugandan *Anopheles* population was very high

**Fig. 5:**
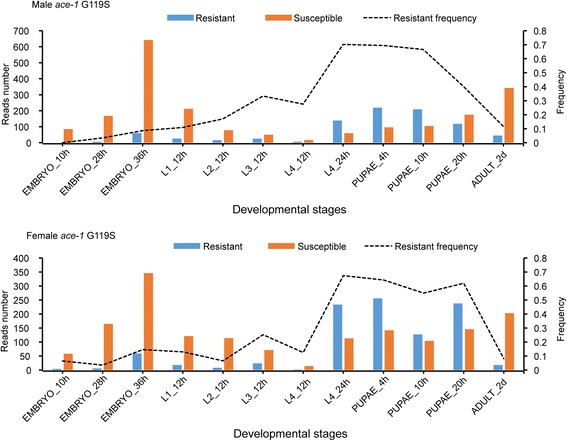
Detection of the G119S mutation in the different sexes of *A. gambiae*. There were no significant differences in the resistance frequency between males and females (t-test, *P* = 0.566). The sequencing depths were different in various samples, the read counts were varied. We recommend using the mutation frequency

**Fig. 6:**
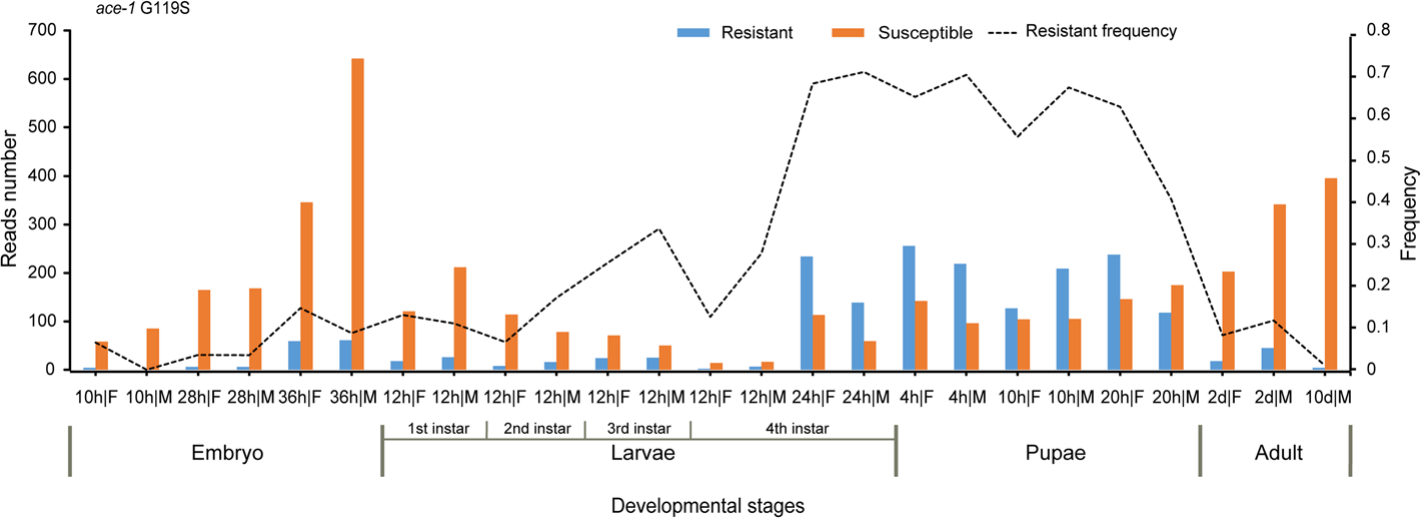
Detection of the G119S mutation in the different developmental stages of *A. gambiae*. The late 4th instar larvae and pupae stages had higher resistance frequencies than the embryo and adult stages (One-way ANOVA test, *p* < 0.01)

We used the ACE pipeline to analyse the RNA-Seq data of 7 insect pests, including *An. gambiae*, *C. floridanus*, *N. vitripennis*, *C. suppressalis*, *P. xylostella*, *N. lugens* and *B. tabaci* (Additional file 3: Table S3). In *An. gambiae*, the major vector of Plasmodium falciparum malaria, we obtained RNA-Seq data from 468 samples, of which 20 were from an eastern Ugandan population. Since the G119S mutation of *ace1* has been reported to confer insecticide resistance, we identified resistant reads from all 468 RNA-Seq data of *An. gambiae* by using the ACE pipeline. The results indicated that the resistance frequency was 30%–44% in the eastern Ugandan population, suggesting that the resistance in the Ugandan *Anopheles* population has reached very high frequency (Fig. 4). There were no significant differences between male and female *An. gambiae* (t-test, *P*-values = 0.566, Fig. 5). Surprisingly, we found significant differences among different developmental stages of the Pimperena strain of *An. gambiae*. The resistance frequency was significantly higher in late larvae and pupae than in the embryo and adult stages (One-way ANOVA test, F = 27.621, *p*-value = 8.186E-7, Fig. 6). The high resistance frequency in the late larvae and pupae stages enables mosquitoes to survive the insecticide treatment. However, mutations often incur high fitness costs such as low fecundity. Our results showed that the mosquito population had a low resistance frequency at the adult stage, thus enabling the mosquitoes to produce offspring with a relatively high fitness. The detail mechanism is worthy of further investigation.

**Fig. 7:**
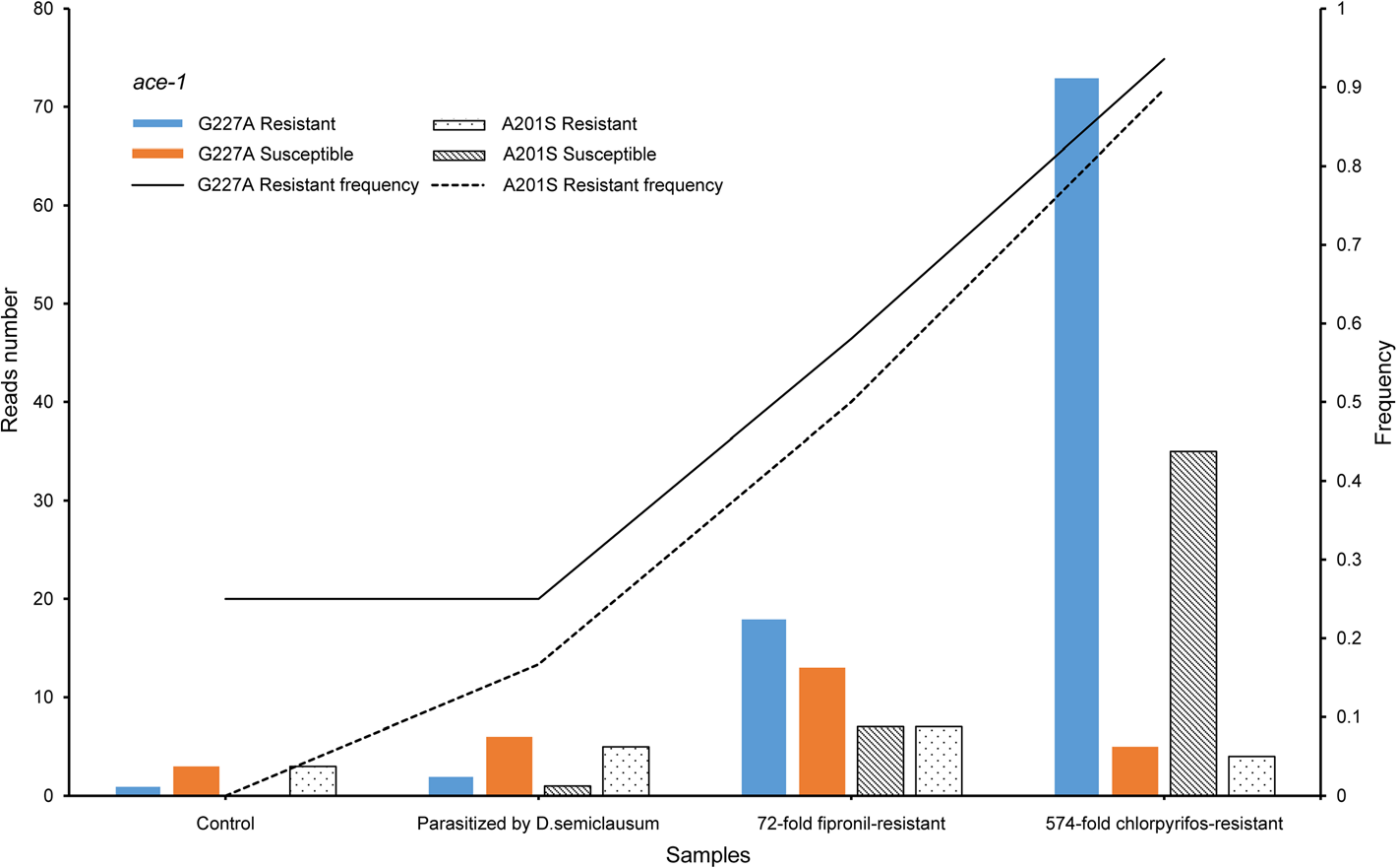
The frequencies of the G227A and A201S mutations in the different samples of *Plutella xylostella*. The G227A mutation was positively associated with resistance to OP or carbamate insecticides, whereas the A201S mutation was not a major contributor

Analyses of the *P. xylostella* RNA-Seq data indicated that the G227A mutation was positively related with resistance levels to organophosphate or carbamate insecticides (F-test, *p* < 0.0.5), whereas the A201S mutation was only a minor contributor (F-test, *p* > 0.0.5, Fig. 7). The wasp *N. vitripennis* and ant *Camponotus floridanus* had a low frequency of resistant reads (<5%, Table 1). However, the agricultural pests *C. suppressalis* and *B. tabaci* had a high resistance frequency. Approximately 70% of *C. suppressalis ace* reads were resistant (Table 1), and most of the *B. tabaci* RNA-Seq data had >90% resistant *ace* reads. All *ace* reads in the 30 *B. tabaci* RNA-Seq data were resistant reads, suggesting that *B. tabaci* has developed extremely high resistance to insecticides (Additional file 4: Table S4).

**Table 1:** The resistance frequencies of predicted from RNA-Seq data by ACE

Species	SRA accession number	Resistance frequency (%)								References
		G118S *ace2*	A201S *ace1*	A201S *ace1*	G227A *ace1*	F290 V *ace1*	F330S *ace1*	F331H *ace1*	S332 L *ace1*	
*Nasonia vitripennis*	SRR1262367					1.9	1.4			(Hoedjes, et al., 2015) [43]
	SRR1262372		3.6			2.1				
	SRR1262376				3.7					
	SRR1262379						2.4			
	SRR940321					1		1.2		(Os, et al., 2013) [44]
	SRR940323	66.7			2.7	0.9				
	SRR1566027							4.8		(Wang, et al., 2015) [45]
*Camponotus floridanus*	SRR1609918				2.5					(Gupta, et al., 2015) [46]
	SRR330970					3.8				(Bonasio, et al., 2012) [47]
	SRR490202				0.9					(Simola, et al., 2013) [48]
*Chilo suppresssalis*	SRR651040			73.5						(Wu, et al., 2013) [49]
	SRR2015503			70.8						(Xu, et al., 2015) [50]
	SRR1200447									(Cao, et al., 2014) [51]

## Discussion

Insecticide resistance is a major problem in agriculture. Target insensitivity induced by mutations has been well studied. In past decades, several target site mutations have been identified in the insect *ace* gene. PCR-based methods have been developed to detect resistance mutations [3, 4, 21]. Recently, RNA-Seq data obtained by using NGS techniques provide a valuable means to study insecticide resistance. Millions of raw reads can be obtained in a single run, thus enabling detection of low frequency mutations. Here, we developed a pipeline, ACE, to identify resistance-associated mutations by using RNA-Seq data. ACE has a high sensitivity and can detect resistant reads at low frequency. It should be noted that very low frequencies of resistant reads should be interpreted with caution due to the possibility of genotyping errors. Owing to the rapid development of NGS techniques, the cost of RNA-Seq has significantly decreased. This pipeline is useful for monitoring resistance-associated mutation(s) in field population by using RNA-Seq data. ACE is also applicable for detecting resistance mutations from the genome re-sequencing data.

The ACE pipeline was used to analyse RNA-Seq data from 7 insect pests. The results proved that the ACE pipeline can successfully detect resistance mutations from millions of reads. Calculating the resistance frequency from the RNA-Seq data of these insect pests confirmed the importance of target site mutations in conferring insecticide resistance. Large-scale level analyses also provided new insights into the evolution of and changes in resistance mutations. We found that the resistance mutation frequency changed during insect development. This change has not been previously reported and is worthy of further investigation.

As a tool to detect resistance-associated mutations from RNA-Seq data, we plan to develop additional integrated applications for ACE to address the following areas. First, development of insecticide resistance is a complex system. Different insecticides have various targets: organophosphate and carbamate insecticides target AChE; pyrethroids insecticides target sodium channels; neonicotinoid insecticides target nicotinic acetylcholine receptors (nAChR); and diamide insecticides target ryanodine receptors (RyR). We wish to broaden the scope of ACE to detect resistance mutations in all target genes. Second, increased metabolism of insecticides, owing to overexpression of detoxification enzymes, is another important mechanism of insecticide resistance. We wish to develop ACE to examine the abundance of P450, GST and esterase genes, which have been reported to have important roles in conferring resistance [22, 23]. Third, cross-resistance provides important information to improve the prediction efficiency [24–27], which has been well studied in human [28, 29], we wish to integrate this information in the future. Last, it has been reported that multiple alterations of gene sequences, such as alternative splicing and RNA editing, are also involved in insecticide resistance. We plan to develop ACE to detect novel SNPs and other types of sequence changes.

## Conclusions

A computational tool was developed to detect insecticide resistance-associated mutation of AChE from insect RNA-Seq data. Both the standalone software and the Web server of ACE were provided. Analyses of 971 RNA-Seq data from 136 studies in 7 insect pests provided new insights into insecticide resistance, suggesting that insecticide resistance mutation might be associate with development stage of insects. Large-scale detection of insecticide resistance mutation using ACE demonstrated that the insecticide resistance of the eastern Ugandan mosquito population and whitefly *B. tabaci* has reached extremely high level.

## Methods

### Data sources

The *ace* sequences were retrieved from GenBank of the National Centre for Biotechnology Information (NCBI) [30]. We selected the *ace* genes of 8 insects as the sequence references. These *ace* were confirmed by using PCR and gene function analysis in the published reports of other groups, including *ace2* in *D. melanogaster* (NP_476953), *ace1* and *ace2* in *Culex tritaeniorhynchus* (BAD06210, BAD06209), *ace1* and *ace2* in *Plutella xylostella* (AAY34743, AAL33820), *ace1* and *ace2* in *Chilo suppressalis* (ABO38111, ABR24230), *ace1* and *ace2* in *Tribolium castaneum* (ADU33189, ADU33190), *ace1* and *ace2* in *Rhopalosiphum padi* (AAT76530, AAU11285), *ace1* and *ace2* in *Aphis gossypii* (AAM94376, AAM94375), and *ace1* and *ace2* in *Liposcelis bostrychophila* (ACN78619, ABO31937). The amino acid sequences of these 15 ACHE were used as the query sequences in BLASTP against the official gene set (OGS) in InsectBase (E-value = 1e–30). The best BLASTP hit was treated as the candidate *ace*. To ensure reliability, sequences less than 1800 bp were removed. All identified ACHEs were confirmed to have two conserved motifs (WIY(F)GGG and FGESAE). These steps yielded 62 *ace1* from 62 species and 70 *ace2* from 70 species (Additional file 1: Table S1).

A total of 971 RNA-Seq data from 136 studies in 7 insect pests (*An. gambiae*, *C. floridanus*, *N. vitripennis*, *C. suppressalis*, *P. xylostella*, *N. lugens* and *B. tabaci*) were downloaded from the Sequence Read Archive database (SRA) of NCBI [31]. The SRA accession numbers are given in Additional file 2: Table S2.

### Phylogenetic analysis

The amino acid sequences of AChE were aligned using MUSCLE [32]. The phylogenetic relationships were inferred using the neighbour-joining method [33] with 1000 replicates. The bootstrap values are shown next to the branches [34]. The evolutionary distances were computed using the Kimura 2-parameter method [35] and expressed as the number of base substitutions per site. The analysis involved 132 nucleotide sequences. All positions containing gaps and missing data were eliminated. There were 1239 positions in the final dataset. A phylogenetic tree was constructed by MEGA 7 [36]. A consensus tree was displayed and edited with iTOL [37]. The tree was drawn to scale, with branch lengths in the same units as those of the evolutionary distances used to infer the phylogenetic tree.

### Collecting known *ace* resistance-associated mutations

To collect the known *ace* resistance-associated mutations, we downloaded the references from NCBI PubMed by searching with the keywords (“insecticide resistance” [Abstract] AND acetylcholinesterase [Abstract]), yielding 440 references. Among these references, only 5 used transcriptome methods to determine *ace* sequences [38–42], and only one reference used raw reads to call SNPs by using SOAPsnp [39]. We manually extracted *ace* mutations conferring insecticide resistance, which yielded 14 mutations at 10 positions in *ace1* and 22 mutations at 18 positions in *ace2*.

## Additional files

Additional file 1: Table S1. The NCBI accession numbers of insect *ace-1* and *ace-2* genes

Additional file 2: Table S2. Resistance mutations in *ace-1* and *ace-2* of insects

Additional file 3: Table S3. The SRA accession numbers of 971 RNA-Seq data used for detecting mutations

Additional file 4: Table S4. The resistance frequency of mutation S331 W in different RNA-Seq data of *Bemisia tabaci*

## Abbreviations

AChE: Acetylcholinesterases
AJAX: Asynchronous JavaScript and XML
CSS: Cascading Style Sheets
nAChR: nicotinic acetylcholine receptors
NCBI: National Center for Biotechnology Information
NGS: Next-generation sequencing
OGS: Official gene set
OP: Organophosphate
PASA: PCR amplification of specific alleles
RNA-Seq: RNA sequencing
RyR: Ryanodine receptors
SNP: Single nucleotide polymorphisms
SRA: Sequence Read Archive database
