# Supplementary material for: ACE: an efficient and sensitive tool to detect insecticide resistance-associated mutations in insect acetylcholinesterase from RNA-Seq data
Source: BMC Bioinformatics. 2017 Jul 10;18:330. doi: 10.1186/s12859-017-1741-6 (PMC5504734; doi:10.1186/s12859-017-1741-6)
Supplement: Supplementary file 1 — The NCBI accession numbers of insect ace-1 and ace-2 genes [file 12859_2017_1741_MOESM1_ESM.docx]

Table S1 The NCBI accession numbers of Insect *ace-1* and *ace-2* genes

| **Order** | **Species** | ***ace1*** | ***ace2*** |
| --- | --- | --- | --- |
| Blattodea | *Blattella germanica* | DQ288249.1 | DQ288847.1 |
| Coleoptera | *Alphitobius diaperinus* | EU086057.1 | EU086056.1 |
|  | *Dendroctonus ponderosae* | DPO004665-RA | Low quality |
|  | *Lasioderma serricorne* | GU211888.1 | Low quality |
|  | *Leptinotarsa decemlineata* | JF343436.1 | JF343437.1 |
|  | *Tribolium castaneum* | NM_001293619.1 | HQ260969.1 |
| Diptera | *Aedes albopictus* | AB218421.1 | AB218420.1 |
|  | *Aedes aegypti* | EF209048.1 | Low quality |
|  | *Anopheles coluzzii* | ACOM033983-TA | Low quality |
|  | *Anopheles darlingi* | NA | ADAC000367-TA |
|  | *Anopheles gambiae* | KC631949.1 | AGAP000466-RA |
|  | *Anopheles stephensi* | ASTE007197-TA | ASTE010565-TA |
|  | *Bactrocera oleae* | NA | AF452052.1 |
|  | *Bactrocera cucurbitae* | NA | XM_011188061.1 |
|  | *Bactrocera dorsalis* | NA | AY155500.1 |
|  | *Ceratitis capitata* | NA | EU130781.1 |
|  | *Cochliomyia hominivorax* | NA | FJ830868.1 |
|  | *Culex pipiens* | AJ489456.1 | AM159193.1 (partial) |
|  | *Culex pipiens pallens* | AY762905.1 | Low quality |
|  | *Culex quinquefasciatus* | XM_001847396.1 | XM_001842175.1 |
|  | *Culex tritaeniorhynchus* | AB122152.1 | AB122151.1 |
|  | *Drosophila simulans* | NA | XM_016176807.1 |

| **Table S1.** Continued | |  |  |
| --- | --- | --- | --- |
| **Order** | **Species** | ***ace1*** | ***ace2*** |
| Diptera | *Drosophila virilis* | NA | XM_002056133.2 |
|  | *Drosophila ananassae* | NA | XM_001953033.2 |
|  | *Drosophila erecta* | NA | XM_001980267.2 |
|  | *Drosophila grimshawi* | NA | XM_001990127.1 |
|  | *Drosophila melanogaster* | NA | NM_057605.5 |
|  | *Drosophila mojavensis* | NA | XM_001998863.2 |
|  | *Drosophila persimilis* | NA | XM_002013876.1 |
|  | *Drosophila sechellia* | NA | XM_002031326.1 |
|  | *Drosophila willistoni* | NA | XM_002072251.2 |
|  | *Drosophila yakuba* | NA | XM_002097460.2 |
|  | *Exorista sorbillans* | NA | HM028669.1 |
|  | *Haematobia irritans* | NA | AY466160.1 |
|  | *Lucilia cuprina* | NA | U88631.1 |
|  | *Mayetiola destructor* | Mdes002098-RA | Low quality |
|  | *Musca domestica* | NA | AF281162.1 |
|  | *Phlebotomus papatasi* | JQ922267.1 | Low quality |
|  | *Stomoxys calcitrans* | NA | HM125963.1 |
| Hemiptera | *Acyrthosiphon pisum* | XM_001948618.2 | XM_001948953.3 |
|  | *Aphis glycines* | JQ349160.1 | Low quality |
|  | *Aphis gossypii* | AF502082.1 | AF502081 |
|  | *Bemisia tabaci* | EF675188.1 | EF675190.1 |
|  | *Cimex lectularius* | JN563927.1 | GU597839.1 |

| **Table S1.** Continued | |  |  |
| --- | --- | --- | --- |
| **Order** | ***Species*** | ***ace1*** | ***ace2*** |
| Hemiptera | *Myzus persicae* | AY147797.1(partial) | AF287291.1 |
|  | *Nephotettix cincticeps* | AB264392.1 | Low quality |
|  | *Nilaparvata lugens* | JQ027700.1(partial) | AJ852420.2 |
|  | *Rhopalosiphum padi* | AY667435.1 | AY707318.1 |
|  | *Schizaphis graminum* | AF321574.2 | NA |
|  | *Sitobion avenae* | AY819704.2 | AY707319.1 |
| Hymenoptera | *Acromyrmex echinatior* | AECH22900-RA | XM_011065851.1 |
|  | *Apis dorsata* | XM_006621490.1 | Low quality |
|  | *Apis florea* | XM_003693203.1 | XM_003694182.1 |
|  | *Apis mellifera* | XM_393751.4 | NM_001040230.1 |
|  | *Athalia rosae* | XM_012396780.1 | XM_012396195.1 |
|  | *Bombus impatiens* | XM_012383753.1 | Low quality |
|  | *Bombus terrestris* | XM_003399342.1 | XM_003401864.1 |
|  | *Camponotus floridanus* | CFLO22383-RA | Low quality |
|  | *Cerapachys biroi* | XM_011339571.1 | XM_011337469.1 |
|  | *Fopius arisanus* | XM_011299719.1 | XM_011298824.1 |
|  | *Harpegnathos saltator* | HSAL21697-RA | Low quality |
|  | *Megachile rotundata* | XM_003699292.1 | XM_003701146.1 |
|  | *Microplitis demolitor* | XM_008554984.1 | XM_008554097.1 |
|  | *Nasonia vitripennis* | XM_008216354.1 | XM_001605518.2 |
|  | *Oomyzus sokolowskii* | Low quality | HM212643.1 |
|  | *Polyrhachis vicina* | Low quality | JF742990.1 |

| **Table S1.** Continued | |  |  |
| --- | --- | --- | --- |
| **Order** | ***Species*** | ***ace1*** | ***ace2*** |
| Hymenoptera | *Vollenhovia emeryi* | XM_012022438.1 | XM_012007191.1 |
|  | *Wasmannia auropunctata* | XM_011701045.1 | Low quality |
| Lepidoptera | *Bombyx mandarina* | EU262633.2 | EU262632.2 |
|  | *Bombyx mori* | NM_001043915.1 | NM_001114641.1 |
|  | *Chilo auricilius* | KF574430.1 | KF574431.1 |
|  | *Chilo suppressalis* | EF453724.1 | EF470245.1 |
|  | *Cnaphalocrocis medinalis* | Low quality | FN538987.1 |
|  | *Cydia pomonella* | DQ267977.1 | DQ267976.1 |
|  | *Danaus plexippus* | DPOGS202180-TA | Low quality |
|  | *Heliconius melpomene* | HMEL013432-TA | Low quality |
|  | *Helicoverpa armigera* | JF894118.1 | JF894119.1 |
|  | *Helicoverpa assulta* | DQ001323.1 | AY817736.1 |
|  | *Manduca sexta* | Msex2.09673-RB | Msex2.05726-RB |
|  | *Melitaea cinxia* | GQ489250.1 | GQ489251.2 |
|  | *Plutella xylostella* | JQ085429.1 | AY061975.1 |
| Phthiraptera | *Pediculus humanus capitis* | AB266615.1 | AB266614.1 |
|  | *Pediculus humanus corporis* | AB266606.1 | AB266605.1 |
| Psoptera | *Liposcelis bostrychophila* | FJ647185.1 | EF362950.1 |
|  | *Liposcelis decolor* | Low quality | FJ647187.1 |
|  | *Liposcelis entomophila* | EU854149.2 | EU854150.1 |
|  | *Liposcelis paeta* | GU214754.1 | Low quality |
| Siphonaptera | *Ctenocephalides felis* | FN645950.1 | FN645951.1 |
