# Supplementary material for: ACE: an efficient and sensitive tool to detect insecticide resistance-associated mutations in insect acetylcholinesterase from RNA-Seq data
Source: BMC Bioinformatics. 2017 Jul 10;18:330. doi: 10.1186/s12859-017-1741-6 (PMC5504734; doi:10.1186/s12859-017-1741-6)
Supplement: Supplementary file 3 — The SRA accession numbers of 971 RNA-Seq data used for detecting mutations [file 12859_2017_1741_MOESM3_ESM.docx]

Table S3 The SRA accession numbers of 986 RNA-seq data used for detecting mutation of insect

| **Species** | **SRA accession numbers** |
| --- | --- |
| *Bemisia tabaci* | SRR039231,SRR039730,SRR059302,SRR062575,SRR066636,SRR066637,SRR211281,SRR306284,SRR306285,SRR316271  SRR453538,SRR453539,SRR453540,SRR453541,SRR453542,SRR453543,SRR453544,SRR453545,SRR499845,SRR499846  SRR499847,SRR609289,SRR609290,SRR609291,SRR611714,SRR611715,SRR835756,SRR835757,SRR835869,SRR1040886  SRR1040887,SRR1040888,SRR1104130,SRR1159208,SRR1159209,SRR1159210,SRR1262400,SRR1262401,SRR1262402  SRR1262403,SRR1523521,SRR1523522,SRR1693230,SRR1693363,SRR1693374,SRR1693416,SRR1930109,DRR018506  SRR1943311,SRR2001504,SRR2168747,SRR2168751,SRR2174309,SRR2174310,SRR2174311,SRR2174312,SRR2174316  SRR2174318,SRR2174322,SRR2174324,SRR2174325,SRR2174326,ERR1079147,ERR1337894,ERR1337895,ERR1337896  ERR1337897,ERR1337898,ERR1337899,ERR1337900,ERR1337901,ERR1337902,ERR1337903,ERR1337904,ERR1337905  ERR1337906,ERR1337907,ERR1337908 |
| *Plutella xylostella* | SRR179062,SRR179508,SRR179509,SRR179510,SRR179511,SRR179587,SRR353499,DRR002645,SRR630488,SRR835315  SRR835316,SRR835317,SRR1197748,SRR1272441,SRR2062280,SRR2062281 |
| *Anopheles gambiae*  *Anopheles gambiae*  *Anopheles gambiae* | ERR476715,ERR476716,ERR476717,ERR476718,ERR476719,ERR476720,ERR489297,ERR489298,ERR489299,ERR489300,ERR489301,ERR489302,ERR489303,ERR489304,ERR489305,ERR489306,ERR505234,ERR505235,ERR505236,ERR505237,ERR505238,ERR505239,ERR505240,ERR537781,ERR537785,ERR537786,ERR537788,ERR537789,ERR537790,SRR1179005,SRR1179006,SRR1179007,SRR1179008,SRR1179009,SRR1179010,SRR1179011,SRR1179012,SRR1525402,SRR1525403,  SRR1586022,SRR1587087,SRR1587088,SRR1587089,SRR1587094,SRR1587095,SRR1587096,SRR1587097,SRR1596453,  SRR1596454,SRR1596455,SRR1596456,SRR1596457,SRR1596458,SRR1596459,SRR1596460,SRR1596461,SRR1596462,  SRR1596463,SRR1596464,SRR1596465,SRR1596466,SRR1596467,SRR1596468,SRR1596469,SRR1596470,SRR1596471,  SRR1596472,SRR1596473,SRR1596474,SRR1596475,SRR1596476,SRR1596477,SRR1596478,SRR1596479,SRR1596480,  SRR1596481,SRR1596482,SRR1596483,SRR1596484,SRR1596485,SRR1596486,SRR1596487,SRR1596488,SRR1596489,  SRR1596490,SRR1596491,SRR1596492,SRR1727555,SRR1727718,SRR1727719,SRR1727720,SRR1727721,SRR1727722,  SRR1727723,SRR1727724,SRR1727725,SRR1727726,SRR1755724,SRR1755727,SRR1755728,SRR1755729,SRR1755730,  SRR1755737,SRR1755738,SRR1755743,SRR1755748,SRR1755749,SRR1755752,SRR1755754,SRR1755755,SRR1755758,  SRR1755759,SRR1755760,SRR1755761,SRR1755763,SRR1755765,SRR1755774,SRR1755785,SRR1755786,SRR1755790,  SRR1755807,SRR1755808,SRR1755825,SRR1755826,SRR1755827,SRR1755828,SRR1755836,SRR1755837,SRR1755838,  SRR1755842,SRR1755858,SRR1755859,SRR1755863,SRR1755864,SRR1755866,SRR1755867,SRR1755871,SRR1755872,  SRR1755875,SRR1755876,SRR1755883,SRR1755884,SRR1755885,SRR1755895,SRR1755896,SRR1755897,SRR1755898,  SRR1755903,SRR1755905,SRR1755906,SRR1755914,SRR1755918,SRR1755924,SRR1755925,SRR1755943,SRR1755948,  SRR1756029,SRR1756033,SRR1756035,SRR1756036,SRR1756037,SRR1756039,SRR1756040,SRR1756043,SRR1756049,  SRR1756053,SRR1756054,SRR1756060,SRR1756061,SRR1756063,SRR1756064,SRR1756074,SRR1756075,SRR1756076,  SRR1756077,SRR1756078,SRR1756079,SRR1756080,SRR1756082,SRR1756085,SRR1756089,SRR1756092,SRR1756095,  SRR1756096,SRR1756099,SRR1756100,SRR1756102,SRR1756103,SRR1756107,SRR1756109,SRR1756110,SRR1756114,  SRR1756116,SRR1756117,SRR1756118,SRR1756122,SRR1756123,SRR1756124,SRR1756128,SRR1756129,SRR1756130,  SRR1756131,SRR1756133,SRR1756134,SRR1756135,SRR1756136,SRR1756140,SRR1756141,SRR1756144,SRR1756145,  SRR1756151,SRR1756152,SRR1756153,SRR1756154,SRR1756157,SRR1756158,SRR1756177,SRR1756179,SRR1756202,  SRR1756203,SRR1756204,SRR1756210,SRR1756215,SRR1756218,SRR1756220,SRR1756223,SRR1756225,SRR1756228,  SRR1756244,SRR1756245,SRR1756246,SRR1756247,SRR1756248,SRR1756252,SRR1756253,SRR1756260,SRR1756261,  SRR1756264,SRR1756266,SRR1756271,SRR1756272,SRR1756273,SRR1756276,SRR1756281,SRR1756282,SRR1756283,  SRR1756286,SRR1756290,SRR1756291,SRR1756295,SRR1756299,SRR1756300,SRR1756303,SRR1756304,SRR1756305,  SRR1756310,SRR1756311,SRR1756315,SRR1756316,SRR1756317,SRR1756318,SRR1756319,SRR1756320,SRR1756322,  SRR1756323,SRR1756324,SRR1756325,SRR1756328,SRR1756329,SRR1756331,SRR1756332,SRR1756333,SRR1756334,  SRR1756337,SRR1756338,SRR1756341,SRR1756343,SRR1756344,SRR1756345,SRR1756346,SRR1756348,SRR1756349,  SRR1756353,SRR1756354,SRR1756355,SRR1756359,SRR1756360,SRR1756364,SRR1756367,SRR1756370,SRR1756374,  SRR1756375,SRR1756379,SRR1756380,SRR1756384,SRR1756385,SRR1756387,SRR1756388,SRR1756389,SRR1756390,  SRR1756391,SRR1756392,SRR1756393,SRR1756394,SRR1756395,SRR1756396,SRR1756397,SRR1756398,SRR1756402,  SRR1756405,SRR1756406,SRR1756409,SRR1756413,SRR1756414,SRR1756416,SRR1756421,SRR1756422,SRR1756423,  SRR1756424,SRR1756425,SRR1756427,SRR1756428,SRR1756432,SRR1756440,SRR1756443,SRR1756452,SRR1756453,  SRR1756454,SRR1756458,SRR1756459,SRR1756460,SRR1756461,SRR1756462,SRR1756463,SRR1756464,SRR1756469,  SRR1756472,SRR1756473,SRR1756475,SRR1756476,SRR1756477,SRR1756478,SRR1756480,SRR1756481,SRR1756484,  SRR1756488,SRR1756491,SRR1756498,SRR1756499,SRR1756504,SRR1763906,SRR1763907,SRR1763908,SRR1764963,  SRR1764967,SRR1764972,SRR1764975,SRR1764976,SRR1764977,SRR1764985,SRR1764988,SRR1764989,SRR1764994,  SRR1764996,SRR1765000,SRR1765001,SRR1765003,SRR1765004,SRR1765005,SRR1765006,SRR1765008,SRR1765009,  SRR1765013,SRR1765016,SRR1765021,SRR1765026,SRR1765027,SRR1765030,SRR1765031,SRR1765037,SRR1765040,  SRR1765045,SRR1765046,SRR1765048,SRR1765049,SRR1765050,SRR1765057,SRR1765058,SRR1765068,SRR1765071,  SRR1765075,SRR1765080,SRR1765081,SRR1765083,SRR1765086,SRR1765087,SRR1765090,SRR1765095,SRR1765098,  SRR1765099,SRR1765102,SRR1765103,SRR1765123,SRR1765129,SRR1765131,SRR1765138,SRR1765139,SRR1765140,  SRR1765142,SRR1765143,SRR1765146,SRR1765152,SRR1765156,SRR1765157,SRR1765204,SRR1765205,SRR1765206,  SRR1765209,SRR1765212,SRR1765213,SRR1765218,SRR1765219,SRR1765223,SRR1765225,SRR1765231,SRR1765233,  SRR1765236,SRR1765237,SRR1765238,SRR1765240,SRR1765241,SRR1765245,SRR1765246,SRR1765249,SRR1765250,  SRR1765251,SRR1765252,SRR1765254,SRR1765255,SRR1765256,SRR1765258,SRR1765262,SRR1805322,SRR1805324,  SRR1805326,SRR1805328,SRR1805330,SRR1805332,SRR1805334,SRR1805341,SRR1805343,SRR1805345,SRR1805347,  SRR1805349,ERR537787,ERR588638,ERR588639,ERR588641,ERR588655,ERR588656,ERR588657,ERR588658,ERR588659,ERR588660,ERR588661,ERR588662,ERR588663,ERR588664,ERR588665,ERR588666,ERR588667,ERR588668,ERR588669,ERR744531,ERR744532,ERR840656,ERR840657,SRR1297285,SRR1298713,SRR1298715,SRR1659854,SRR1659855,  SRR1659856,SRR1659857,SRR1659915,SRR1724076,ERR537787,ERR537782,ERR537784,SRR513203,SRR513204,  SRR513205,SRR513206,SRR520427,ERR588643,SRR520428,SRR535750,ERR588644,ERR440790,SRR953402,SRR953451,SRR953486,SRR953487,ERR588645,SRR958820,SRR958821,SRR958822,SRR958823,SRR958824,ERR440792,SRR958825,ERR588646,SRR958826,ERR440793,SRR958827,ERR588647,ERR588648,ERR440788,SRR1171958,ERR588649,  SRR1171976,ERR588650,SRR1172036,ERR588640,ERR440789,ERR588641,SRR1172037,ERR588642,ERR588653,  ERR588654,SRR037071,SRR513189,SRR513190,SRR513191,SRR513192,SRR513193,SRR513194,SRR513195,SRR513196,SRR513197,SRR513198,SRR513199,SRR513200,SRR513201,SRR513202,ERR588651,ERR588652,ERR440791,ERR537778,ERR537783,ERR537779,ERR537780 |
| *Chilo suppresssalis* | SRR449559,SRR651040,SRR1200447,SRR1200448,SRR2015503 |
| *Camponotus floridanus* | SRR059754,SRR059755,SRR059756,SRR059757,SRR059758,SRR330970,SRR330971,SRR330974,SRR330975,SRR490201  SRR490202,SRR490203,SRR490204,SRR490205,SRR490206,SRR1609918,SRR1609919,SRR2050466,SRR2050467  SRR2050468,SRR2050469,SRR2050470,SRR2050471,SRR2050472,SRR2050473,SRR2050474,SRR2050475,SRR2050476  SRR2050477,SRR2050478,SRR2050479,SRR2050480,SRR2050481,SRR2050482,SRR2050483,SRR2050484,SRR2050485  SRR2050486,SRR2050522,SRR2050523,SRR2050524,SRR2050525,SRR2050526,SRR2050527,SRR2050528,SRR2050529  SRR2050530,SRR2050531,SRR2050532,SRR2050533,SRR2050534,SRR2050535,SRR2050536,SRR2050537,SRR2060721  SRR2060722,SRR2060723 |
| *Nasonia vitripennis*  *Nasonia vitripennis* | SRR1566022,SRR1566023,SRR1566024,SRR1566025,SRR1566026,SRR1566027,SRR2748824,SRR2748825,SRR2748826  SRR2748827,SRR2748828,SRR2748829,SRR2748830,SRR2748831,SRR2748832,SRR2748833,SRR2748834,SRR2748835  SRR2748836,SRR2748837,SRR2748838,SRR2748839,SRR2748840,SRR2748841,SRR2748842,SRR2748843,SRR2748844  SRR2748845,SRR2748846,SRR2748847,SRR2748848,SRR2748849,SRR2748850,SRR2748851,SRR2748852,SRR2748853  SRR2748854,SRR2748855,SRR2748856,SRR2748857,SRR2748858,SRR2748859,SRR2748860,SRR2748861,SRR2748862  SRR2748863,SRR2748864,SRR2748865,SRR2748866,SRR2748867,SRR2748868,SRR2748869,SRR2748870,SRR2748871  SRR2748872,SRR2748873,SRR2748874,SRR2748875,SRR2748876,SRR2748877,SRR2748878,SRR2748879,SRR2748880  SRR2748881,SRR2748882,SRR2748883,SRR2748884,SRR2748885,SRR2748886,SRR2748887,SRR2748888,SRR2748889  SRR2748890,SRR2748891,SRR2748892,SRR2748893,SRR2748894,SRR2748895,SRR2748896,SRR2748897,SRR2748898  SRR2748899,SRR2748900,SRR2748901,SRR2748902,SRR2748903,SRR2748904,SRR2748905,SRR2748906,SRR2748907  SRR2748908,SRR2748909,SRR2748910,SRR2748911,SRR2748912,SRR2748913,SRR2748914,SRR2748915,SRR2748916  SRR2748917,SRR2748918,SRR2748919,SRR2748920,SRR2748921,SRR2748922,SRR2748923,SRR2748924,SRR2748925  SRR2748926,SRR2748927,SRR2748928,SRR2748929,SRR2748930,SRR2748931,SRR2748932,SRR2748933,SRR2748934  SRR2748935,SRR2748936,SRR2748937,SRR2748938,SRR2748939,SRR2748940,SRR2748941,SRR2748942,SRR2748943  SRR2748944,SRR2748945,SRR2748946,SRR2748947,SRR2748948,SRR2748949,SRR2748950,SRR2748951,SRR2748952  SRR2748953,SRR2748954,SRR2748955,SRR2748956,SRR2748957,SRR2748958,SRR2748959,SRR2748960,SRR2748961  SRR2748962,SRR2748963,SRR2748964,SRR2748965,SRR2748966,SRR2748967,SRR2748968,SRR2748969,SRR2748970  SRR2748971,SRR2748972,SRR2748973,SRR2748974,SRR2748975,SRR2748976,SRR2748977,SRR2748978,SRR2748979  SRR2748980,SRR2748981,SRR2748982,SRR2748983,SRR2748984,SRR2748985,SRR2748986,SRR2748987,SRR2748988  SRR2748989,SRR2748990,SRR2748991,SRR2773794,SRR2773795,SRR2773796,SRR2773797,SRR2773798,SRR2773799  SRR3438974,SRR3439007,SRR3439008,SRR3457431,SRR3457433,SRR3457434,SRR3748957,SRR3748958,SRR3748959  SRR3748960,SRR3748961,SRR3748963,SRR646520,SRR646521,SRR646522,SRR646523,SRR646524,SRR646525  SRR646526,SRR646527,SRR646528,SRR646529,SRR646530,SRR646531,SRR646532,SRR646533,SRR646534,SRR646535,SRR647669,SRR940321,SRR940323,SRR988300,SRR988301,SRR988302,SRR988303,SRR1262366,SRR1262367  SRR1262368,SRR1262369,SRR1262370,SRR1262371,SRR1262372,SRR1262374,SRR1262375,SRR1262376,SRR1262378  SRR1262379 |
| *Nilaparvata lugens* | SRR064533,SRR871556,SRR1002947,SRR1003049,SRR1187936,SRR1269581,DRR016172,DRR016171,SRR1537484  SRR1537486,SRR1573316 |
